# Multisensory effects on somatosensation: a trimodal visuo-vestibular-tactile interaction

**DOI:** 10.1038/srep26301

**Published:** 2016-05-20

**Authors:** Mariia Kaliuzhna, Elisa Raffaella Ferrè, Bruno Herbelin, Olaf Blanke, Patrick Haggard

**Affiliations:** 1 Center for Neuroprosthetics, School of Life Science, Ecole Polytechnique Fédérale de Lausanne, Lausanne, Switzerland; 2 Laboratory of Cognitive Neuroscience, Brain Mind Institute, School of Life Science, Ecole Polytechnique Fédérale de Lausanne, Lausanne, Switzerland; 3 Institute of Cognitive Neuroscience, University College London, London, United Kingdom; 4 Department of Neurology, University Hospital, Geneva, Switzerland; 5 Department of Psychology, Royal Holloway University of London, Egham, United Kingdom

## Abstract

Vestibular information about self-motion is combined with other sensory signals. Previous research described both visuo-vestibular and vestibular-tactile bilateral interactions, but the simultaneous interaction between all three sensory modalities has not been explored. Here we exploit a previously reported visuo-vestibular integration to investigate multisensory effects on tactile sensitivity in humans. Tactile sensitivity was measured during passive whole body rotations alone or in conjunction with optic flow, creating either purely vestibular or visuo-vestibular sensations of self-motion. Our results demonstrate that tactile sensitivity is modulated by perceived self-motion, as provided by a combined visuo-vestibular percept, and not by the visual and vestibular cues independently. We propose a hierarchical multisensory interaction that underpins somatosensory modulation: visual and vestibular cues are first combined to produce a multisensory self-motion percept. Somatosensory processing is then enhanced according to the degree of perceived self-motion.

Detecting self-motion, and maintaining postural stability require combination of vestibular with visual and somatosensory signals, such as retinal optic flow, shifts of body weight and the quality of body contact with the supporting surface^1^,^2^,^3^,^4^. Visuo-vestibular integration underlies perception of whole body rotations and translations^5^,^6^, even when visual and vestibular stimuli are in conflict^7^,^8^,^9^.

Caloric (CVS) and galvanic (GVS) vestibular stimulation, and natural vestibular stimulation from passive whole-body rotations all increase tactile sensitivity in healthy participants^10^,^11^,^12^. CVS and GVS also transiently improve tactile deficits in neurological patients^13^,^14^,^15^,^16^. Anatomically, visual, vestibular and somatosensory signals converge at the level of the vestibular nuclei^17^, at the thalamus^18^,^19^ and in multisensory cortical regions such as the parietal operculum and the posterior insula^20^,^21^,^22^,^23^.

Despite the close anatomical and behavioural connections between visual and vestibular cues on the one hand, and vestibular and tactile on the other, the *trimodal interaction* between them remains unexplored. In particular, it is unclear whether vestibular-tactile perceptual interactions are merely a by-product of anatomical convergence in the cortex, or instead depend on perceptual representation of environmental self-motion.

Here we investigated visuo-vestibular-tactile interactions in healthy volunteers. Specifically, we explored whether the vestibular effect on touch is a direct consequence of vestibular stimulation or whether it rather depends on prior integration of vestibular and visual signals forming a self-motion representation, that subsequently influences touch (Fig. 1b). Participants detected faint tactile stimuli delivered to either their left or right index fingers in three conditions: a static baseline condition, during passive whole-body rotation (vestibular condition), and during passive whole-body rotation in the presence of visual optic flow (visuo-vestibular condition). Crucially, vestibular input was identical in the two rotation conditions, but the perceived speed of self-motion was reduced in the condition where concurrent optic flow was present (i.e., an optic flow signalling slower velocity in the opposing direction, so that during leftward chair rotation, congruent flow direction would involve dots flowing towards the right). This indicates that visuo-vestibular signals are effortlessly integrated, building a percept of self-motion^9^,^24^. If the influence of vestibular signals on tactile sensitivity is a by-product of an anatomical vestibular-somatosensory convergence, we should observe identical vestibular modulations of touch in both rotation conditions, whether visual motion is present or not (Fig. 1b). If, however, tactile sensitivity is modulated by an integrated visuo-vestibular signal then tactile enhancement should be reduced in the visual-vestibular condition, relative to the vestibular alone, due to the effect of optic flow on self-motion (Fig. 1b).

## Materials and Methods

### Participants

All participants were naïve to the goals of the experiment. They were reimbursed at the rate of 20 CHF per hour. The study was conducted in line with the Declaration of Helsinki. The experimental protocol was approved by the local ethics committee (École Polytechnique Fédérale de Lausanne). The methods were carried out in accordance with the approved guidelines. Participants gave written informed consent in advance. All participants were right-handed as assessed by informal verbal inquiry.

### Procedure

Four experiments were conducted. Experiments 1 and 2 were designed to select combinations of visual and vestibular stimulation for which optic flow would most strongly influence perceived rotation speed. Experiment 3 compared the effects on tactile sensitivity of combined visuo-vestibular rotational stimuli with those of vestibular rotational stimuli alone. Experiment 4 controlled for possible effects of visual optic flow alone on tactile sensitivity, in the absence of vestibular signals.

### Experimental setup

#### The same experimental setup was used in all four experiments

Participants were seated inside a sound-shielded dark room in a custom-built centrifuge cockpit-style chair which delivered passive, whole-body, rotational stimuli. Head and body motion were prevented by using head fixation, a restraining harness and cushioning. The chair was digitally servo-controlled (National Instrument PCI-7352) with precision of around 0.1°. The chair rotated in the yaw plane and was centred on the rotation axis, thus delivering only angular acceleration vestibular stimuli. The rotation profiles of the chair were pre-set, and constituted 1000 ms of acceleration to a given speed followed by 1000 ms deceleration to a stationary position in either clockwise or counter clockwise direction. Trials were separated by 5000 or 6000 ms of no rotation. Participants fixated a central cross on a head-mounted display. The beginning of a trial was signalled by a change in colour of the fixation cross (changed from white to red). The head-mounted display displayed a 3D pattern of moving dots (optic flow), generated by in-house software ExpyVR. The optic flow consisted of a 3D pattern of randomly distributed white dots, size – 25 pixels, placed at different depths, the movement of which followed a raised cosine profile. Rotation was simulated by placing the subject’s viewpoint in the middle of the scene and rotating it around the yaw axis. The motion of the chair and the motion of the optic flow were synchronised in time. In the vestibular alone condition, where no optic flow was presented, participants fixated a central point.

#### Experiment 1: Influences on perceived self-motion speed during chair rotation

**Figure 1:**
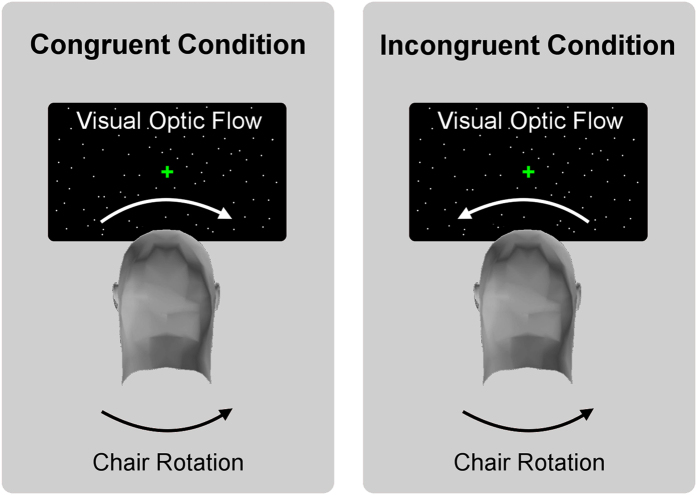
Experiment 1 and 2: conditions. Experimental conditions for Experiment 1 and 2. Participants were seated in the rotating chair wearing a head-mounted display showing a pattern of moving dots. Optic flow was presented in the naturally congruent direction (i.e., opposing direction, so that during leftward chair rotation, congruent flow direction would involve dots flowing towards the right). In the naturally incongruent condition, vestibular and visual rotations are in the same external direction (e.g., during leftward chair rotation, incongruent flow direction involves dots flowing towards the left).

This experiment aimed to investigate (a) how perceived self-motion speed depended on actual chair rotation velocity (b) whether visual optic flow could influence perceived speed of self-motion during chair rotation, and (c) how velocity and direction of visual and vestibular stimuli combined to influence perceived self-motion speed. Participants (15, mean age 24.04 years, SD = 4.8 years, 4 females) used a joystick to mark the perceived speed of self-motion on a scale from 0 to 100. At the beginning of the experiment they were exposed to four 100°/s rotations (two clockwise, two counter clockwise) as the maximal reference point, and then completed 4 training trials with different chair rotation and optic flow velocity combinations. During the rotation participants fixated a central point. A horizontal scale showing only the extreme points of 0 and 100 appeared after each rotation and stayed on the screen for 5 seconds. On every trial the cursor of the scale appeared at 0 (left end). Three vestibular speeds were used (30, 60, 90°/s). In each case, optic flow was presented at the same speed, in the naturally congruent direction (i.e., opposing direction, so that during leftward chair rotation, congruent flow direction would involve dots flowing towards the right, see Fig. 1).

For example, a 30°/s vestibular rotation was paired with 30°/s congruent optic flow, 60°/s vestibular rotation was paired with 60°/s optic flow, etc. This combination was designed to simulate a rotating chair in a static visual environment (recall that the optic flow was delivered via a head-mounted display, so congruent optic flow at chair speed would correspond to the experience of rotating in a static room). In addition, to investigate subtler effects of optic flow speed on perceived self-motion speed, we presented two further visual stimuli at each vestibular velocity: a slow-rate 10°/s optic flow in a congruent direction, and a 10°/s optic flow in the incongruent direction. There were thus nine experimental conditions each repeated 20 times. The experiment was divided into four short blocks (~5 min each). The same number of clockwise and counter clockwise rotations was used.

#### Experiment 2: Effects of optic flow velocity on perceived self-motion speed during 90°/s chair rotation

Experiment 2 fixed the chair rotation speed at its experimental value of 90°/s, and investigated how different optical flow speeds and directions might modulate perceived self-motion speed. Participants (14, mean age 26.1 years, SD = 4.2 years, 2 females) were exposed to vestibular rotation at 90°/s in conjunction with each of four visual speeds (10, 30, 45 and 90°/s), in either congruent or incongruent directions (the 90°/s speed was presented only in the congruent direction). A vestibular-only condition, without optic flow, was also tested. There were thus eight conditions repeated 20 times.

Experiments 1 and 2 were preliminary stimulus selection procedures, designed to identify optimal parameters for visual modulation of perceived self-motion speed caused by vestibular stimulation during chair rotation. Using these data, we separately investigated whether changes in optic flow rate and direction influenced the perceived speed of self-motion as predicted by a simple linear model of combined visual and vestibular velocity signals:


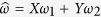


Where 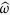 is the participant’s velocity estimate, *ω*_1_ and *ω*_2_ are the vestibular and the visual velocities produced by the chair and the optic flow, respectively, and X and Y are factors susceptible to influence subjective perception of these velocities (neural and physical noise, amount of conflict between the modalities, or different weights attributed to each sense)^25^.

**Figure 2:**
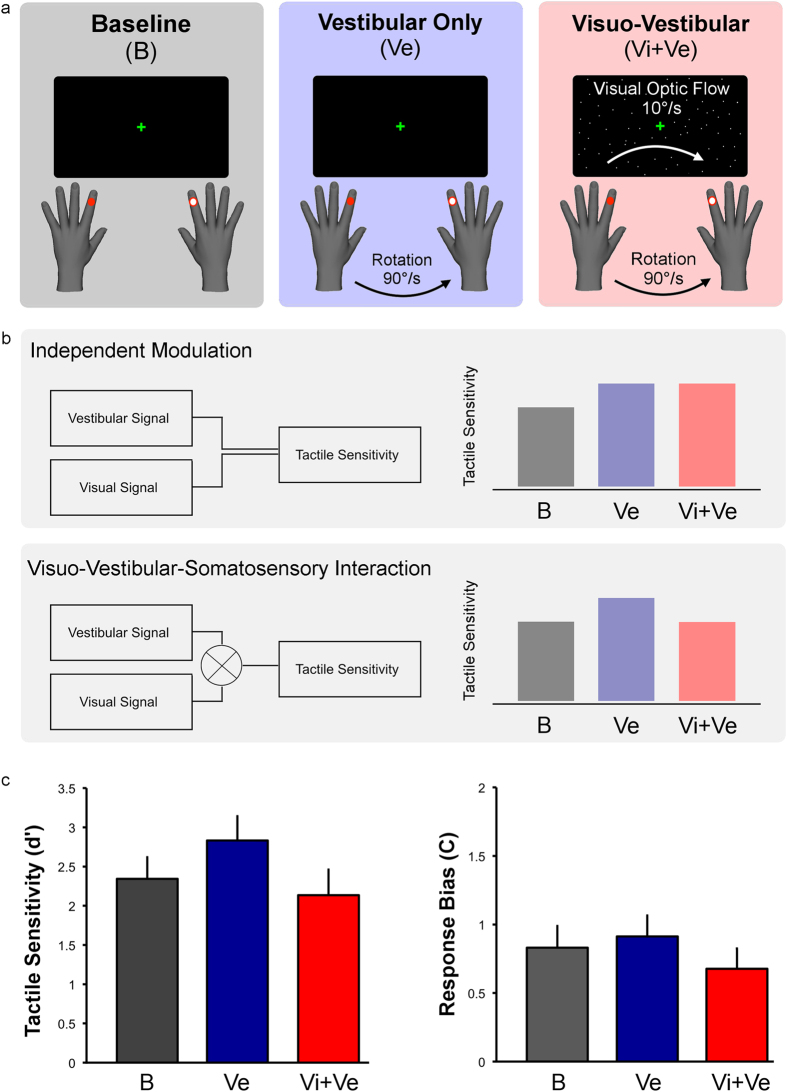
Experiment 3: conditions and results. (**a**) Experimental conditions for Experiment 3. Participants were seated in the rotating chair wearing a head-mounted display showing (or not) a pattern of moving dots. Participants were asked to detect faint tactile stimuli delivered to their right or left index fingers (black colour indicates stimulus present). Three conditions were tested: no rotation baseline (B), vestibular only condition (Ve, passive whole-body rotations at 90°/s) and visuo-vestibular condition (Vi+Ve, passive whole-body rotation at 90°/s associated with speed incongruent optic flow at 10°/s). (**b**) Experimental hypothesis. If the influence of vestibular signals on tactile sensitivity is a direct product of the activation of the vestibular projections, data should show an increase in somatosensory sensitivity in both Ve and Vi + Ve conditions (independent modulation hypothesis). Conversely, if somatosensory sensitivity is affected by integrated visual and vestibular signals leading to the perception of slower speed, tactile enhancement should be reduced in the Vi + Ve condition, relative to Vi (visuo-vestibular-somatosensory interaction). (**c**) Sensitivity (d’) and response bias (C) data as a function of experimental condition. Results show higher sensitivity in the vestibular only condition as opposed to the baseline and visuo-vestibular conditions. No difference was found between the latter two. There were no significant differences in response bias. Error bars represent the standard error.

Experiments 1 and 2 jointly produced results that guided selection of vestibular and visual stimuli for our main experiments. We found that increasing optic flow velocity increased perceived self-motion speed. The effect of *congruent* optic flow on perceived self-motion speed was indeed monotonic, and as predicted by the linear model. For example, if the chair is rotating at 90°/s to the right, higher velocities of congruent (i.e., leftward) optic flow result in a higher perceived speed of self-motion. In contrast, the linear model predicts that higher velocities of incongruent optic flow should result in a lower perceived speed of self-motion (e.g.^25^ Fig. 2b). However, this prediction for incongruent optic flow was not fulfilled (see discussion for possible interpretations). Across both experiments, we consistently showed that congruent optic flow at 10°/s indeed reduced perceived self-motion speed relative to higher velocities of congruent optic flow, as predicted by a simple linear model for combination of visual and vestibular signals. We thus chose congruent 10°/s optic flow and 90°/s chair speed (see^12^) as the most convincing combination to dissociate purely vestibular from combined visuo-vestibular signals regarding self-motion speed. These parameters were therefore used for the main Experiments 3 and 4.

#### Experiment 3: Combined visual and vestibular effects on tactile sensitivity

We investigated two hypotheses regarding vestibular and visuo-vestibular effects on touch (Fig. 1b). First, a hypothesis of direct vestibular–somatosensory interaction would predict similar effects of vestibular input on tactile sensitivity whether visual input was simultaneously present or not. That is, there should be similar modulation of tactile sensitivity in vestibular and visuo-vestibular conditions. Alternatively, we might hypothesise that somatosensation is modulated by a self-motion signal based on combined visuo-vestibular information. This view predicts that congruent visuo-vestibular stimulation should influence tactile sensitivity less than purely vestibular stimulation generated by chair rotation alone.

Participants (14, mean age 25.1 years, SD = 3.7 years, 5 females) were asked to detect faint tactile stimuli delivered to the distal phalanxes of their left and right index fingers by solenoid tappers in different experimental conditions^12^. Stimulation intensity was manually adjusted in the following way. A staircase procedure was used to identify the lowest intensity at which a tactile stimulus could be reliably detected on each finger. Stimuli of increasing intensity were applied until participants reported a sensation. Stimulus intensity was successively decreased and then increased again until exactly 5 of 10 stimuli were detected. Next, the intensity obtained was tested in an automated detection block (24 trials: 4 signal-absent, 10 left finger stimuli, 10 right finger stimuli in randomised order) to check that 40–60% of stimuli were reliably detected. This level was taken as a working estimate for near-threshold tactile stimulation and used during the experiment.

Our design factorially combined passive body rotation, optic flow and tactile stimulation conditions. Every trial involved a single rotation (if present), during which a single shock (if present) would be delivered. In particular, we presented three experimental conditions: (i) A no rotation static *baseline condition*, in which the shock was delivered either to the left or right index finger without whole-body rotation; (ii) a *Vestibular condition*, in which the shock was delivered during 90°/s yaw whole body rotation, (iii) a *Visuo-vestibular condition*, in which the shock was delivered during 90°/s yaw rotation combined with optic flow of 10°/s in the congruent direction (Fig. 1a).

The tactile detection task was designed using a signal detection approach^26^; for each condition, the stimulus was present in 30 trials and absent in another 30 trials. In the vestibular and visuo-vestibular conditions, 16 tactile stimuli were delivered to the right hand (half during clockwise and half during counter-clockwise rotation), and 14 tactile stimuli to the left hand (half during clockwise and half during counter clockwise rotation). For the signal-absent trials, 15 clockwise and 15 counter-clockwise rotations were presented, but no tactile stimulation occurred. In the baseline condition 15 tactile stimuli were delivered to the left hand and 15 to the right hand, and no chair rotation occurred. Participants thus performed a total of 180 tactile detection trials, divided into five blocks, and presented in a randomised order. Before each block, the tactile detection threshold was checked and adjusted if required.

The presence or absence of the stimulus, the hand stimulated and the direction of rotation were unpredictable. Participants were asked to fixate a fixation cross throughout the block. The tactile stimuli, when present, occurred 700 ms after the beginning of the trial, signalled by the change in colour of the fixation cross from green to red. This latency corresponded to peak rotation velocity for the rotation trials. During the baseline and vestibular conditions, the HMD showed only the fixation cross, for a duration of 2000 ms. Participants had 4000 ms to verbally report whether they felt the tactile stimulus (“yes”) or not (“no”). That is, in the rotation conditions the rotational stimulation lasted for 2000 ms, followed by 4000 ms response time, and an additional 2000 ms rest. During the experiment white noise was presented over the participants’ headphones and a black blanket covered the chair, to avoid participants’ inferring the rotation direction from external auditory or visual cues. Data for each trial were recorded and analysed later.

#### Experiment 4: Effects of optic flow alone on tactile sensitivity, without vestibular stimulation

This experiment controlled for direct effects of optic flow on tactile sensitivity, in the absence of vestibular stimulation. Tactile sensitivity was tested in two conditions: with and without 10°/s optic flow stimulation. Participants (14, mean age 25.2 years, SD = 3.6 years, 2 females) were placed in the rotating chair, which was always stationary but was powered on as in Experiments 1–3. The optic flow used was the same as in Experiment 3. On half the trials the flow simulated clockwise rotation, and on the other half counter-clockwise rotation. Participants performed a total of 120 trials; for each condition the tactile stimulus was present in 30 trials and absent in the other 30 trials. The experiment was divided into three blocks.

## Results

### Experiment 1: Does visual information influence perceived self-motion speed during chair rotation?

Perceived rotation speed data are shown in Table 1. First, we performed main-effect planned comparisons as a manipulation check, to verify that perceived self-motion speed indeed varied monotonically with chair rotation velocity. For these comparisons we averaged across the different optic flow conditions, and performed a Bonferroni correction for 2 comparisons (30°/s vs 60°/s, and 60°/s vs 90°/s), thus setting the significance level to 0.025. Overall, 30°/s chair speed rotations were indeed judged as slower than 60°/s chair speed rotations (t(14) = −7.453, p < 0.001), which were judged slower than 90°/s chair speed rotations (t(14) = −9.628, p < 0.001). Thus, the vestibular stimulation provided by chair rotation strongly influenced perceived self-motion speed.

The combinations of vestibular and visual conditions were interpreted using the predictions of a simple linear model of visual-vestibular speed perception. This model predicts that both the speed and the direction of optic flow might influence perceived self-motion speed. In particular, the model predicts that (a) increasing the velocity of a congruent optic flow during chair rotation should increase the perceived rotation speed, and (b) an incongruent optic flow should produce a lower perceived rotation speed than a congruent optic flow with the same rate. As the design of experiment 1 was not fully factorial, we used a series of planned comparisons, rather than ANOVA, to test the predictions. Specifically, prediction (a) and prediction (b) were tested separately at each of the three rotation velocities, resulting in six planned comparisons. We therefore applied a Bonferroni correction with a significance threshold of 0.05/6, i.e., 0.00833. The probability values are reported uncorrected, but only results beyond the corrected threshold were interpreted.

To test prediction (a), we compared the 10°/s congruent flow condition with the conditions involving congruent optic flow at velocities matching the chair rotation velocities, i.e., 30°/s, 60°/s, and 90°/s respectively within each chair speed condition. All three comparisons were significant even after Bonferroni correction (t(14) = −7.819, p < 0.001, t(14) = −8.108, p < 0.001, and t(14) = −4.784, p < 0.001 respectively), consistent with a combination of congruent visual and vestibular stimuli. To test prediction (b), we compared the perceived self-motion speed for congruent vs incongruent 10°/s optic flow conditions. After Bonferroni correction, no significant differences were found between congruent and incongruent optic flow when chair speed was set at 30°/s (t(14) = −2.141, p = 0.050), 60°/s (t(14) = −1.272, p = 0.224) or 90°/s (t(14) = 2.778, p = 0.015).

### Experiment 2: Identification of optic flow velocity that maximally influences perceived self-motion speed during 90°/s chair rotation

Perceived rotation speed data are shown in Table 2. As the design of Experiment 2 was not fully factorial, we again used planned comparisons, and we again separately investigated the predictions of changing optic flow rate, and of changing optic flow direction. We performed a total of 8 planned comparisons, so we used a Bonferroni correction, adjusting the significance level to 0.05/8 = 0.00625. Probability values are shown prior to correction.

We first investigated whether changes in optic flow rate influenced perceived self-motion speed in the manner predicted by a simple linear model of visual-vestibular combination. In the case of congruent optic flow, we indeed found that faster optic flow rates lead to higher perceived self-motion speeds as predicted by the linear model, (though this just failed to reach the corrected value for significance for the comparison of 10°/s vs 30°/s: t(13) = −3.188, p = 0.007, 30°/s vs 45°/s: t(13) = −3.331, p = 0.005). In addition, 90°/s congruent optic flow was judged as significantly faster than the no flow condition (t(13) = 3.370, p = 0.005), again as predicted by the linear model.

In the case of incongruent optic flow, we found that faster optic flow led to higher perceived self-motion speeds, although the linear model in fact predicts an effect in the opposite direction (10°/s vs 30°/s: t(13) = −5.364, p < 0.001, 30°/s vs 45°/s: t(13) = −3.923, p = 0.002). We also compared the effects of optic flow direction at each optic flow speed. We found no significant differences at lower optic flow rates, but a significant difference in the opposite direction from the linear model prediction at the highest optic flow rate (congruent 10°/s vs incongruent 10°/s: t(13) = 0.743, p = 0.471, congruent 30°/s vs incongruent 30°/s (t(13) = −2.803, p = 0.015, congruent 45°/s vs incongruent 45°/s: t(13) = −3.762, p = 0.002, with the incongruent condition judged as faster).

The results for congruent optic flow largely confirmed those of experiment 1, and were consistent with a linear visuo-vestibular combination. In contrast, incongruent optic flow results were again contrary to the model prediction. Simple linear combination of vestibular and incongruent visual signals should, in principle, *reduce* perceived self-motion speed, with higher optic flow rates producing a stronger reduction. However, Table 2 shows that (a) incongruent and congruent optic flow produced very similar perceived self-motion speeds, and (b) increasing the rate of incongruent optic flow always produced an *increase* in perceived self-motion speed, rather than the decrease predicted by a model of linear visuo-vestibular combination. In fact, the pattern of judgements for incongruent optic flow suggests that participants reported only visual speed, and did not base their reports of rotation speed on a simple linear combination of visual and vestibular signals. In effect, the percept of visual motion seemed to over-ride the percept of self-motion. Therefore, we avoided incongruent visual stimulation in the main experiment.

Based on the results of experiments 1 and 2, we selected 10°/s congruent optic flow as the visual stimulation of experiment 3 and 4, for two reasons. First, 10°/s congruent optic flow results were always consistent with a simple model of visual-vestibular combination. Second, 10°/s congruent optic flow always produced the maximum dissociation in perceived self-motion speed between a purely vestibular and a visual-vestibular condition.

### Summary Experiments 1 & 2

The combination of visual and vestibular signals in computing self-motion speed has been studied extensively^25^,^27^,^28^,^42^. Interestingly, most of these studies focused on whether vestibular stimulation could alter a visually-induced perception of vection, most used continuous stimulation over much longer epochs than those studied here, and most asked participants to report the directional, vector quantity of velocity, rather than the scalar quantity of speed. In contrast, we have focussed on whether visual stimulation can modulate a transient vestibular percept of speed. Nevertheless, three results of that literature are particularly relevant here. First, the contribution of optic flow to perceived self-motion typically emerged only after several seconds of stimulation^28^. Second, when vestibular and visual signals are in conflicting directions (as in our incongruent condition), the normal linear combination of cues breaks down, and a dramatic switch towards weighting a single cue may occur^28^. Third, the results on visual or vestibular dominance in such conflict conditions appear to vary across studies^25^,^27^,^28^. Fourth, all studies agree that visual information dominates at lower frequencies, while vestibular information dominates at higher frequencies. For example, in a condition similar to our incongruent flow condition, Zacharias and Young^25^ found that vestibular signals initially lead to a high perceived velocity, with incongruent visual signals later producing a reduction in perceived velocity (see their figure 10 d). Our short epochs and fast chair rotation speeds may have encouraged a strong vestibular contribution to perceived self-motion.

We speculate that, when incongruent optic flow was presented, participants may have strongly weighted the visual stimulus, with a low weighting for the vestibular signal. The reasons for this remain unclear. However, the current literature does not identify a unique set of weighting functions for vestibular and visual signals contributing to self-motion, but rather notes that weightings depend strongly on directional congruence, and on frequency. Visual dominance in the perception of self-motion has been reported in some previous studies^7^,^8^. The detailed pattern of these interactions, and the factors that influence the weighting of vestibular and visual stimuli await further research. However, preliminary experiments 1 and 2 yielded two results. First, congruent optic flow with simultaneous chair rotation indeed produced a percept of self-motion reflecting a linear visuo-vestibular combination. Second, a slow-rate congruent optic flow combined with a fast chair rotation provided the strongest dissociation between purely vestibular and visuo-vestibular percepts of self-motion. We therefore used these findings to select visual and vestibular signals used in our main experiments investigating combined visuo-vestibular effects on tactile sensitivity.

### Experiment 3: Combined visual and vestibular effects on tactile sensitivity

Signal detection analysis was applied to the tactile detection results, allowing us to extract perceptual sensitivity (d’) and response bias (C) estimates for each participant and condition. These values were subjected to an ANOVA comparing the three experimental conditions (baseline, vestibular and visuo-vestibular). The main effect of experimental conditions was found to be significant for sensitivity values (F(2, 26) = 3.6895, p = 0.039; *η*^*2*^ = 0.22). Two tailed post-hoc t-tests showed significantly better sensitivity in the vestibular condition (mean d’ = 2.83, SD = 1.16) compared with the baseline condition (mean d’ = 2.34, SD = 1.04) (t(13) = −2.28, p = 0.04; Cohen’s d = 0.45), no difference between baseline condition and the visuo-vestibular (mean d’ = 2.14, SD = 1.22) condition (t(13) = 0.71, p = 0.5), and significantly better sensitivity in the vestibular condition compared with the visuo-vestibular condition (t(13) = 2.53, p = 0.03; Cohen’s d = 0.59). No correction for multiple comparisons is required for post-hoc tests following a significant omnibus ANOVA with three conditions^29^. No significant differences were found for the response bias (F(2, 26) = 1.3616, p = 0.3) (Fig. 1c) (mean values: baseline 0.83, SD = 0.59; vestibular 0.91, SD = 0.57; visuo-vestibular 0.68, SD = 0.55).

### Experiment 4: Independent effects of optic flow on tactile sensitivity

Sensitivity and response bias were estimated for each experimental condition (Table 3). Two tailed t-tests showed no significant difference between the baseline and optic flow conditions for neither sensitivity (t(13) = 0.033, p = 0.97) nor response bias (t(13) = 0.77, p = 0.46).

## Discussion

The tentacular nature of cortical projections from the peripheral vestibular organs may explain the numerous interactions that vestibular signals have with other sensory modalities. The combination of visual, vestibular and tactile information underlies detection of self-motion^30^,^31^,^32^,^33^, postural stability^34^,^35^ and spatial orientation^36^,^37^,^38^,^39^. Recent behavioural, neuropsychological and psychophysiological studies have confirmed the importance of both close visuo-vestibular interactions^5^,^6^,^40^, and of vestibular-tactile interactions^10^,^11^,^12^,^13^,^41^.

We found that the effect of yaw rotation on touch was reduced in the visuo-vestibular condition, compared to a vestibular only condition. Thus, when speed-incongruent visual and vestibular signals were combined (producing a slower perception of self-motion), tactile sensitivity deteriorated relative to a vestibular only condition, and was no longer enhanced relative to baseline. Thus, in the visuo-vestibular condition, tactile sensitivity was significantly worse than in the vestibular alone condition, despite identical yaw rotations in these two conditions. Two explanations could account for these results. On the one hand, tactile sensitivity could be influenced by an integrated percept of visual and vestibular stimuli. On the other hand, vestibular and visual information could independently and simultaneously affect tactile sensitivity. In this case an effect of optic flow alone on tactile sensitivity should be observed: in particular, 10°/s optic flow stimuli (as tested here) should modulate tactile sensitivity. Experiment 4 did not find any evidence to support this prediction, and thus ruled out the possibility that optic flow has an independent effect on tactile sensitivity. More specifically, we found no effect of 10°/s optic flow on tactile sensitivity, as compared to a baseline without rotation or optic flow.

Our results indicate that the integration of visual and vestibular signals to produce a self-motion percept does not always follow a simple linear summation of the two sensory cues. Interestingly, the earlier studies that formed the basis of the linear combination hypothesis^25^,^28^,^42^ used very different vestibular stimuli to ours, typically involving sustained rotations over several seconds. In contrast, we wanted to produce a transient vestibular afferent signal, to investigate possible event-related modulations of somatosensation. We found no evidence in the literature of linear summation for such transient signals. We therefore speculate that the brief nature of our stimuli could explain the nonlinear patterns of visual-vestibular interaction that we observed.

Our study also suggests that self-motion may influence tactile sensitivity in a non-linear fashion, having an effect only when a certain magnitude of perceived self-motion is achieved. The linearity of vestibular-tactile interactions has not been systematically explored. One previous study shows that somatosensory enhancement is triggered by even very brief galvanic vestibular stimulations. Further, the degree of tactile sensitivity enhancement was independent of the latency and dose of stimulation^53^. This suggests an “all-or-nothing” mechanism, whereby any magnitude of galvanic stimulation above a certain threshold level would produce the same enhancement in tactile sensitivity. The present results might reflect a similar all-or-nothing mechanism for effects of self-motion on tactile sensitivity. Our 10°/s congruent optic flow might have reduced the self-motion signal below the level required to trigger enhanced tactile sensitivity.

Our results demonstrate that somatosensory processing in the presence of a visuo-vestibular combination is not driven by the vestibular afferent signal directly, nor by two independent inputs from visual and vestibular organs. Our result also rules out the possibility that somatosensory facilitation is just due to a non-specific factor of stimulus-evoked arousal. An account based on arousal would predict stronger somatosensory facilitation in the visuo-vestibular condition than in the vestibular alone condition, because of the additional visual stimulation. In fact, we found a significant effect in the opposite direction. Instead, somatosensory detection is driven by the integrated visuo-vestibular stimulus that specifies *self-motion*. Importantly, this result contrasts with a recent experiment involving arbitrary, non-natural coincidence of visual and vestibular events^44^. In that experiment, we investigated whether visual flashes from an LED, or brief near-infrared caloric vestibular stimuli, or the combination of both events, would influence tactile sensitivity. We found that visual and vestibular stimuli had independent influences on tactile sensitivity, with no interaction when the two stimuli were presented simultaneously. In that experiment, there was no natural integration of visual and vestibular stimuli into a single percept, since visual and vestibular stimuli were arbitrary and unrelated. For example, the visual and vestibular stimuli could not be ascribed to any single common event, such as a head motion. In the present experiment, in contrast, visual and vestibular stimuli were selected because they could be successfully integrated into a single self-motion percept. The contrast between the present experiment using natural rotation and the previous experiment using artificial vestibular and visual stimuli^44^ highlights the crucial assumption of a common source event that underlies visual-vestibular integration.

The anatomical locus of both the visual-vestibular interaction, and the multisensory-tactile interaction remains speculative, because our experiment was purely behavioural. In contrast, the neural basis of *visual*-vestibular interactions for self-motion has been studied extensively. Some neurons that integrate both vestibular stimulation and optic flow are found as early in the processing stream as the vestibular nuclei and the thalamus^17^,^18^. Visual, vestibular and tactile signals thereafter overlap at multiple levels. Vestibular neurons in the thalamus also respond to tactile stimulation on the animal’s paw^19^. In the cerebral cortex, vestibular-somatosensory interactions were found in the intraparietal sulcus, and in the primary somatosensory cortex^22^,^23^,^43^,^45^. Human neuroimaging studies reported similar convergence of vestibular and somatosensory projections^46^,^47^,^48^. The parietal cortex also hosts visuo-somatosensory interactions^23^. Finally, trimodal visuo-vestibular-tactile neurons were found in the parietal regions (ventral intraparietal area, VIP; parietoinsular vestibular cortex, PIVC) of non-human primates^23^,^49^,^50^. Those studies reported trimodal neurons with tactile receptive fields on the head and face, but VIP also receives a large number of hand and finger projections^51^,^52^. Interestingly, neurons responding to both visual and vestibular stimulation were reported more frequently than vestibular-tactile cells^23^,^49^,^50^. Nevertheless, integrated visuo-vestibular percepts could clearly influence tactile sensitivity at any of these several levels.

It is possible that the visuo-vestibular-tactile interplay we observe here would differ for tactile stimuli directly related to self-motion and balance, e.g. on the soles of the feet, on the face and neck, or on the fingertips when the hands are in contact with a stable environmental surface. In those situations, in contrast to the present experiment, tactile information could potentially be integrated into the self-motion percept. The anatomical substrate of this interplay has yet to be identified.

In the meantime, we show that the combination of visual and vestibular cues signalling self-motion can significantly influence tactile sensitivity. This result sheds light on the possible functions of the vestibular-tactile interaction reported previously. We found that variations in somatosensory perception were better explained by an influence of a self-motion percept, rather than by ‘raw’ vestibular signals. An animal navigating around its environment is likely to come in contact with environmental objects, and will need to respond to them with appropriate approach or withdrawal actions. Heightened tactile sensitivity during self-motion would facilitate such responses.

## Additional Information

**How to cite this article**: Kaliuzhna, M. *et al*. Multisensory effects on somatosensation: a trimodal visuo-vestibular-tactile interaction. *Sci. Rep.* **6**, 26301; doi: 10.1038/srep26301 (2016).

## Figures and Tables

**Table 1:** Experiment 1 Results.

Condition		Subjectively judged speed (VAS)	
Vestibular speed	Optic flow speed	mean	SD
30°/s	congruent 10°/s	21.1	8.9
	incongruent 10°/s	22.9	11.3
	congruent 30°/s	32.4	16.3
60°/s	congruent 10°/s	38.1	10.0
	incongruent 10°/s	39.3	11.1
	congruent 60°/s	57.4	16.2
90°/s	congruent 10°/s	67.7	9.8
	incongruent 10°/s	64.6	11.2
	congruent 90°/s	80.8	11.4

Means and standard deviations reported for each vestibular speed (30°/s, 60°/s, 90°/s) in conjunction with 10°/s congruent or incongruent optic flow. A condition with optic flow congruent with the vestibular speed was also tested.

**Table 2:** Experiment 2 Results.

Condition		Subjectively judged speed (VAS)	
Vestibular speed	Optic flow speed	mean	SD
90°/s	congruent 10°/s	55.1	14.7
	congruent 30°/s	62.9	12.3
	congruent 45°/s	67.6	11.2
	incongruent 10°/s	54.1	15.3
	incongruent 30°/s	66.2	9.5
	incongruent 45°/s	73.1	10.4
	congruent 90°/s	82.1	11.2
	no flow	68.6	13.4

Means and standard deviations reported for each optic flow condition.

**Table 3:** Experiment 4 Results.

Condition	Sensitivity		Response Bias	
	mean	SD	mean	SD
Baseline	2.16	0.98	0.96	0.57
Visual alone	2.15	1.07	0.83	0.57

Mean sensitivity and response bias for each experimental condition.
